# Growth and tolerance of infants fed formula supplemented with polydextrose (PDX) and/or galactooligosaccharides (GOS): double-blind, randomized, controlled trial

**DOI:** 10.1186/1475-2891-11-38

**Published:** 2012-06-07

**Authors:** Claude Ashley, William H Johnston, Cheryl L Harris, Suzanne I Stolz, Jennifer L Wampler, Carol Lynn Berseth

**Affiliations:** 1 Alabama Clinical Therapeutics, 364 Honeysuckle Road, Dothan, AL, 36305, USA; 2 Birmingham Pediatric Group, 806 St Vincent’s Drive, Birmingham, AL, 35205, USA; 3 Clinical Research, Department of Medical Affairs, Mead Johnson Nutrition, Evansville, IN, 47721, USA

**Keywords:** Infant formula, Galactooligosaccharides, Polydextrose, Prebiotics

## Abstract

**Background:**

To ensure the suitability of an infant formula as the sole source of nutrition or provide benefits similar to outcomes in breastfed infants, advancements in formula composition are warranted as more research detailing the nutrient composition of human milk becomes available. This study was designed to evaluate growth and tolerance in healthy infants who received one of two investigational cow’s milk-based formulas with adjustments in carbohydrate, fat, and calcium content and supplemented with a prebiotic blend of polydextrose (PDX) and galactooligosaccharides (GOS) or GOS alone.

**Methods:**

In this multi-center, double-blind, parallel-designed, gender-stratified prospective study 419 infants were randomized and consumed either a marketed routine cow’s milk-based infant formula (Control; Enfamil® LIPIL®, Mead Johnson Nutrition, Evansville, IN) (n = 142) or one of two investigational formulas from 14 to 120 days of age. Investigational formulas were supplemented with 4 g/L (1:1 ratio) of a prebiotic blend of PDX and GOS (PDX/GOS; n = 139) or 4 g/L of GOS alone (GOS; n = 138). Anthropometric measurements were taken at 14, 30, 60, 90, and 120 days of age. Daily recall of formula intake, tolerance, and stool characteristics was collected during study weeks 1 and 2 and 24-h recall was collected at 60, 90, and 120 days of age. Medically-confirmed adverse events were recorded throughout the study.

**Results:**

There were no group differences in growth rate from 14 to 120 days of age. Discontinuation rates were not significantly different among study groups. No differences in formula intake or infant fussiness or gassiness were observed. During study weeks 1 and 2 and at 60 days of age stool consistency ratings were higher (i.e. softer stools) for infants in the PDX/GOS and GOS groups versus Control and remained higher at 120 days for the PDX/GOS group (all *P* < 0.05). The overall incidence of medically-confirmed adverse events was similar among groups.

**Conclusions:**

Investigational routine infant formulas supplemented with 4 g/L of either a prebiotic blend of PDX and GOS or GOS alone were well-tolerated and supported normal growth. Compared to infants who received the unsupplemented control formula, infants who received prebiotic supplementation experienced a softer stooling pattern similar to that reported in breastfed infants.

**Trial registration:**

ClinicalTrials.gov Identifier: NCT00712608

## Introduction

To ensure the suitability of an infant formula as the sole source of nutrition or provide benefits similar to outcomes in breastfed infant populations [1], adjustments in level of fat (including levels of long-chain polyunsaturated fatty acids, or LCPUFAs), protein, carbohydrate, vitamins and minerals, iron, electrolytes, or other optional ingredients may be warranted as the composition of human milk is better defined. In particular, human milk oligosaccharides (HMOs) [2,3] are the third largest component of human milk (5–10 g/L in mature milk) after lactose and fat [4] and comprise a class of carbohydrates considered to be bifidogenic. HMOs modulate the infant immune system as well as influence the development of the intestinal microbiota [5]. Prebiotics share functional attributes with HMOs and are defined as “a selectively fermented ingredient that allows specific changes, both in the composition and/or activity in the gastrointestinal microbiota that confers benefits upon host well-being and health [6].” These beneficial effects include but are not limited to the following: a) increased beneficial bacteria (e.g. bifidobacteria); b) decreased pathogenic bacteria; and c) an improved laxation pattern (e.g. softer stools) [7-11].

In healthy term infants, we previously demonstrated that a cow’s milk-based infant formula supplemented with a new prebiotic blend of polydextrose (PDX) and galactooligosaccharides (GOS) (1:1 ratio at a level of 4 g/L) was well-tolerated, supported normal growth, promoted a stool consistency closer to that of breastfed infants, [9] and produced soft stools and a bifidogenic effect closer to breast milk when compared to infants fed an unsupplemented formula [12] indicating that the PDX and GOS blend meets the European Society for Paediatric Gastroenterology Hepatology and Nutrition (ESPGHAN) and the Food and Agriculture Organization of the United Nations (FAO) definition of a prebiotic (ie., confers a health benefit on the host associated with modulation of the microbiota) [13,14]. In the present study, we evaluated the effect of infant formulas with adjustments in fat, carbohydrate, and calcium composition as well as supplementation with 4 g/L of the prebiotic blend of PDX and GOS or GOS alone on overall growth and tolerance in healthy term infants from 14 to 120 days of age.

## Methods

### Study population

Healthy 12- to 16-day old infants were recruited at 21 clinical sites in the United States. Eligible infants were singleton births at 37–42 weeks gestational age with birth weight ≥ 2500 g and solely formula-fed at least 24 h prior to randomization. Exclusion criteria included history of underlying disease or congenital malformation likely to interfere with normal growth and development or participant evaluation; feeding difficulties or formula intolerance; weight at randomization <98% of birth weight; large for gestational age (defined as birth weight-for-age exceeding 90th percentile [15,16] born from a mother who was diabetic at childbirth; and immunodeficiency.

### Study design

In this multicenter, double-blind, randomized, controlled, parallel-group, prospective trial, participants were enrolled between July 2008 and April 2009. The objective was to evaluate growth and tolerance in healthy, term infants. The study sponsor created a computer-generated, gender-stratified randomization schedule provided in sealed consecutively-numbered envelopes for each study site. Study formula was assigned by opening the next sequential envelope from the appropriate set at the study site. Study formulas, each designated by two unique codes known only to the sponsor, were dispensed to parents at each study visit prior to completion or withdrawal. Neither the product labels nor the sealed envelopes allowed direct unblinding by the study site. Personnel responsible for monitoring the study were also blinded to study product identification. Blinding for a participant could be broken by study sponsor personnel in the event of a medical emergency in which knowledge of the study formula was critical to the participant’s management. In this study, it was not necessary to break the study code prematurely.

Participants were randomly assigned to receive a marketed routine cow’s milk-based infant formula (Control; Enfamil LIPIL, Mead Johnson Nutrition, Evansville, IN) or one of two investigational formulas from 14 to 120 days of age. All study formulas were provided in powdered form and could not be differentiated by smell, consistency or any other characteristics; identical mixing instructions were provided to yield a final product of 20 calories/fluid ounce. All study formulas were supplemented with docosahexaenoic acid (DHA) at 17 mg/100 kcal. The investigational formulas differed from the Control in total fat, total carbohydrate, level of arachidonic acid (ARA), and calcium source (Table 1). Investigational formulas were also supplemented with 4 g/L (1:1 ratio) of a blend of PDX (Litesse® Two Polydextrose; Danisco) and GOS (Vivinal® GOS Galactooligosaccharide; Friesland Foods Domo) (PDX/GOS) or 4 g/L of GOS alone (GOS).

**Table 1:** Nutrient composition per 100 kcal

**Nutrient**	**Study Formula (target values)**		
	**Control**	PDX/GOS*	**GOS†**
Total Protein, g‡	2.1	2.1	2.1
Total Fat, g§	5.3	5.6	5.6
Linoleic acid, mg	860	900	900
α-Linolenic acid, mg	85	85	85
ARA, mg	34	25	25
DHA, mg	17	17	17
Total Carbohydrate, g||	10.9	10.6	10.6
Vitamin A, IU	300	300	300
Vitamin D, IU	60	60	60
Vitamin E, IU	2	2	2
Vitamin K, mcg	9	9	9
Thiamin, mcg	80	80	80
Riboflavin, mcg	140	140	140
Vitamin B6, mcg	60	60	60
Vitamin B12, mcg	0.3	0.3	0.3
Niacin, mcg	1000	1000	1000
Folic Acid, mcg	16	16	16
Pantothenic Acid, mcg	500	500	500
Biotin, mcg	3	3	3
Vitamin C, mg	12	12	12
Choline, mg	24	24	24
Inositol, mg	6	6	6
Calcium, mg¶	78	78	78
Phosphorus, mg	43	43	43
Magnesium, mg	8	8	8
Iron, mg	1.8	1.8	1.8
Zinc, mg	1	1	1
Manganese, mcg	15	15	15
Copper, mcg	75	75	75
Iodine, mcg	15	15	15
Selenium, mcg	2.8	2.8	2.8
Sodium, mg	27	27	27
Potassium, mg	108	108	108
Chloride, mg	63	63	63

* Supplemented with prebiotic blend of PDX and GOS at 4 g/L.

† Supplemented with GOS at 4 g/L.

‡ Sources of protein: non-fat dry milk and whey protein concentrate.

§Sources of fat: base blend of palm olein, soybean, coconut, and high oleic sunflower oils; DHA from algal oil and ARA from fungal oil.

||Sources of carbohydrate: Control, 10.9 g lactose; PDX/GOS, 10.0 g lactose and 0.6 g prebiotic oligosaccharides; GOS, 10.0 g lactose and 0.6 g prebiotic oligosaccharides.

¶Sources of calcium: In all study formulas, 66% of calcium was derived from non-fat dry milk and whey protein concentrate. In the Control, the remaining 34% was derived from calcium carbonate. In both the PDX/GOS and GOS, the remaining 34% was derived from calcium gluconate.

### Ethics

Parents or guardians provided written informed consent prior to enrollment. The research protocol and informed consent forms observing the Declaration of Helsinki (including October 1996 amendment) were approved by the institutional review board/ethics committee of each participating institution. The study complied with good clinical practices.

### Study objectives and outcomes

Anthropometric measures (body weight, length, and head circumference) were recorded at study visits corresponding to enrollment (14 ± 2 days), 30 (±3 days), 60 (±3 days), 90 (±3 days), and 120 (±4 days) days of age. Parents completed a baseline 24-h recall of intake (fluid oz/day), tolerance (fussiness and gassiness), and stool characteristics (frequency and consistency) at study enrollment and daily recall was obtained during the initial 14 days of the study (study weeks 1 and 2) beginning the evening of study randomization. A 24-h recall of diet, tolerance, and stool characteristics was collected at 60, 90, and 120 days of age. Responses were scaled from 0 to 3 for amount of gas (none, slight amount, moderate amount, excessive amount); 0 to 4 for fussiness (not fussy, slightly fussy, moderately fussy, very fussy, extremely fussy); and 1 to 5 for stool consistency (hard, formed, soft, unformed or seedy, watery). The primary outcome was weight growth rate from 14 to 120 days of age. Secondary outcomes included other anthropometric and tolerance measures and medically-confirmed adverse events. Adverse events were coded according to specific event (e.g. otitis media, colic, etc.) and the body system involved including: Body as a Whole; Cardiovascular; Eye, Ears, Nose, and Throat; Gastrointestinal; Metabolic and Nutrition; Musculoskeletal; Respiratory; Skin; and Urogenital.

### Statistical methods

The sample size was chosen to detect a clinically relevant difference of 3 g/day in weight gain from 14 to 120 days of age (80% power; one-tailed). Assuming a standard deviation of 6 g/day for male and 5 g/day for female participants, approximately 78 males and 55 females were needed to enroll in each group with the expectation that 51 male and 36 female participants per study group would complete the study. Analysis of variance (ANOVA) was used to assess growth rates from 14 to 30, 60, 90, or 120 days of age calculated by fitting a linear regression model to each participant's data. The dependent variable was the growth measurement; the independent variable was the actual days of age of the participant. The slope from the linear model was the growth rate. Mean weight growth rates by gender for each investigational formula group were compared with the control using one-tailed tests as outlined in guidance provided by the American Academy of Pediatrics (AAP) Task Force on Clinical Testing of Infant Formulas [17]. Due to differences detected in body weight for males at enrollment, “body weight at enrollment” was included as a covariate for both males and females in the statistical model to analyze weight growth rate. For all secondary outcomes, overall comparisons for the three formula groups were two-tailed. Unadjusted pairwise comparisons were performed if the overall test was statistically significant. All tests were conducted at α = 0.05. Achieved weight, length, and head circumference; length and head circumference growth rates; formula intake, and stool frequency were analyzed by ANOVA. Stool consistency, fussiness, and gas were analyzed using the Cochran-Mantel-Haenszel (CMH) row means score test. Incidence of adverse events was analyzed using Fisher’s exact test. To be included in analysis of a specific outcome at each measured time point, participant data was required to be collected within a specific range, usually ±7 days of the study visit. All analyses were conducted using SAS version 9.1 (Cary, NC).

## Results

### Participants

**Figure 1:**
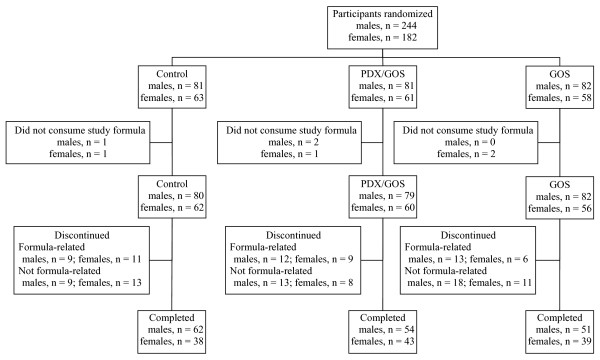
Flow of study participants.

A total of 426 participants were enrolled and randomized (Control: 144; PDX/GOS: 142; GOS: 140). Participants who were randomized but consumed no study formula (Control: 2; PDX/GOS: 3; GOS: 2) were not included in subsequent analyses (Figure 1). The population analyzed was comprised of all infants randomized to one of the study formulas who received at least one feeding. No differences in body weight, length, or head circumference were observed by gender among groups at study enrollment with the exception of body weight in males (Table 2). Birth anthropometric measures as well as gender, race, and ethnic distribution were also similar among groups (data not shown). No statistically significant group differences were detected for study discontinuation (Control: 42, 30%; PDX/GOS: 42, 30%; GOS: 48, 35%) or discontinuation related to study formula (Control: 20, 14%; PDX/GOS: 21, 15%; GOS: 19, 14%). In the total study population, 53 participants (13%) discontinued due to formula intolerance as determined by the study investigator; the most common symptoms were fussiness (Control: 13; PDX/GOS: 10; GOS: 9), gas (Control: 10; PDX/GOS: 4; GOS: 7), and vomiting (Control: 7; PDX/GOS: 3; GOS: 9). Parental decision was the most common reason for discontinuation unrelated to study formula (56 participants, 13%). A total of 287 infants completed the study (Control: 100; PDX/GOS: 97; GOS: 90).

**Table 2:** Infant characteristics at study entry Infant characteristics at study entry

	**Study Group**		
	**Control**	**PDX/GOS**	**GOS**
Total number of participants	142	139	138
Number of males/females	80/62	79/60	82/56
males^*^			
Weight (g)	3784.1 ± 47.0	3798.9 ± 47.3	3648.4 ± 46.4†
Length (cm)	52.7 ± 0.2	53.0 ± 0.2	52.6 ± 0.2
Head circumference (cm)	36.4 ± 0.1	36.4 ± 0.1	36.2 ± 0.1
females^*^			
Weight (g)	3598.5 ± 53.9	3603.2 ± 54.8	3582.3 ± 56.7
Length (cm)	52.1 ± 0.2	52.2 ± 0.2	51.8 ± 0.3
Head circumference (cm)	35.7 ± 0.1	35.9 ± 0.1	35.6 ± 0.2

^*^ Mean ± standard error (SE).

† Significantly lower vs. Control or PDX/GOS, *P* < 0.05.

### Growth

**Figure 2:**
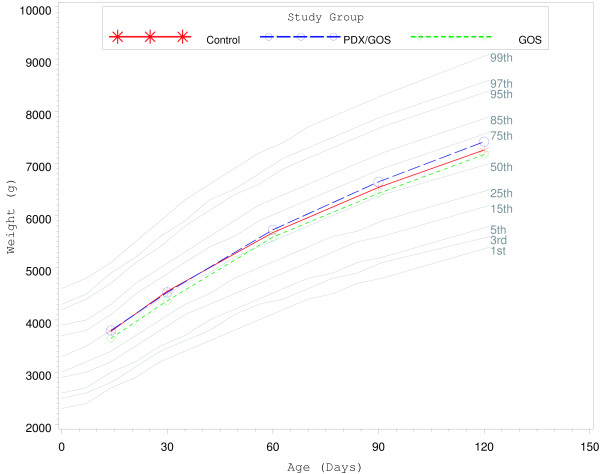
** Mean achieved weight for male participants with World Health Organization (WHO) reference percentiles (1**^**st**^**to 99**^**th**^**) from 14 to 120 days of age.** Control, stars; PDX/GOS, circles; GOS, diamonds.

**Figure 3:**
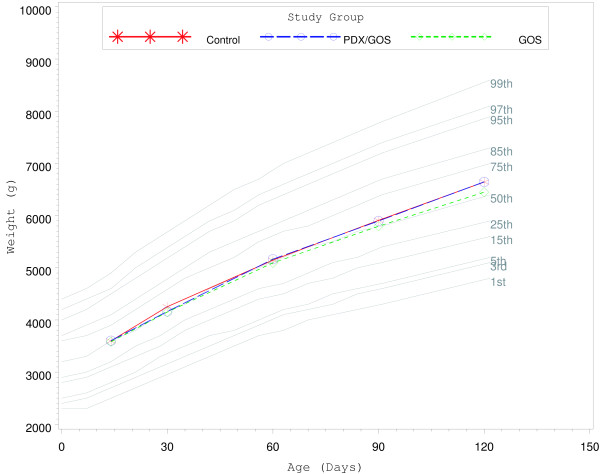
** Mean achieved weight for female participants with World Health Organization (WHO) reference percentiles (1**^**st**^**to 99**^**th**^**) from 14 to 120 days of age.** Control, stars; PDX/GOS, circles; GOS, diamonds.

From day 14 to 30 weight growth rate was significantly lower for males in the PDX/GOS vs. Control group and for females in the GOS vs. Control group (*P* < 0.05; Table 3). However, no other significant differences were observed for weight, length, or head circumference growth rates by gender for any age range among study groups. In addition, no significant group differences were observed for mean achieved weight, length, or head circumference at any measured time point. Finally, mean achieved weight for males (Figure 2) and females (Figure 3) plotted on the WHO weight-for-age standard growth chart [18,19] fell between the 25^th^ and 75^th^ percentiles.

**Table 3:** Weight, length, and head circumference growth rates from 14 days to 30, 60, 90, and 120 days of age

**Gender**	**Day**	**Group (n)**	Growth rate*		
			**Weight† (g/day)**	**Length (cm/day)**	**Head circumference (cm/day)**
male	30	Control (74)	48.9 ± 1.5	0.15 ± 0.009	0.10 ± 0.005
		PDX/GOS (68)	45.2 ± 1.5‡	0.15 ± 0.009	0.10 ± 0.005
		GOS (68)	46.7 ± 1.6	0.13 ± 0.009	0.10 ± 0.005
	60	Control (66)	41.3 ± 1.1	0.13 ± 0.004	0.08 ± 0.002
		PDX/GOS (59)	41.4 ± 1.2	0.13 ± 0.004	0.07 ± 0.002
		GOS (60)	41.9 ± 1.2	0.13 ± 0.004	0.07 ± 0.002
	90	Control (62)	36.1 ± 1.0	0.12 ± 0.003	0.07 ± 0.002
		PDX/GOS (55)	37.4 ± 1.0	0.12 ± 0.003	0.06 ± 0.002
		GOS (52)	36.8 ± 1.1	0.12 ± 0.003	0.06 ± 0.002
	120	Control (63)	32.6 ± 0.8	0.11 ± 0.002	0.06 ± 0.001
		PDX/GOS (54)	34.0 ± 0.9	0.11 ± 0.002	0.06 ± 0.001
		GOS (50)	33.3 ± 0.9	0.11 ± 0.002	0.06 ± 0.001
female	30	Control (52)	38.4 ± 1.5	0.15 ± 0.012	0.09 ± 0.006
		PDX/GOS (50)	34.9 ± 1.6	0.12 ± 0.012	0.08 ± 0.007
		GOS (49)	34.6 ± 1.6‡	0.11 ± 0.012	0.09 ± 0.007
	60	Control (43)	32.4 ± 1.2	0.12 ± 0.005	0.07 ± 0.003
		PDX/GOS (44)	32.1 ± 1.2	0.12 ± 0.005	0.06 ± 0.003
		GOS (42)	32.2 ± 1.3	0.12 ± 0.006	0.07 ± 0.003
	90	Control (40)	29.0 ± 1.0	0.11 ± 0.003	0.06 ± 0.002
		PDX/GOS (43)	29.7 ± 0.9	0.11 ± 0.003	0.05 ± 0.002
		GOS (38)	29.3 ± 1.0	0.11 ± 0.003	0.06 ± 0.002
	120	Control (38)	27.6 ± 0.9	0.10 ± 0.003	0.05 ± 0.002
		PDX/GOS (44)	27.9 ± 0.8	0.10 ± 0.002	0.05 ± 0.001
		GOS (39)	27.3 ± 0.9	0.10 ± 0.003	0.05 ± 0.002

^*^ Mean ± standard error (SE).

† Adjusted for body weight at enrollment.

‡ Significantly lower than Control, *P* < 0.05, one-tailed test.

### Tolerance

At enrollment, parent-reported gassiness and fussiness (data not shown) and stool characteristics (Table 4) were similar among groups. No significant group differences in gassiness, fussiness, or study formula intake were detected during study weeks 1 or 2, or at 60, 90, or 120 days of age. For the initial two weeks of feeding, means for amount of gas were low (≤1.6, between slight amount and moderate amount of gas on the gassiness scale) and mean levels of fussiness were also low (≤1.6, between slightly fussy and moderately fussy on the fussiness scale) in all groups. Using 24-hour recall at 60, 90, and 120 days of age, amount of gas most commonly reported was slight amount or moderate amount and fussiness was most often characterized as slightly fussy or not at all fussy in all groups. Mean (±SE) stool frequency (number/day) was significantly lower in the Control versus PDX/GOS or GOS groups during study week 1 (2.2±0.2 vs 3.7±0.2 or 3.9±0.2; all *P* < 0.05) and study week 2 (2.2±0.2 vs 3.6±0.2 or 3.7±0.2; all *P* < 0.05). This pattern continued through 60 days of age, but by 90 days of age, no statistical differences in stool frequency were detected between study groups (Table 4). Mean (±SE) stool consistency (with categories corresponding to 1 = hard, 2 = formed, 3 = soft, 4 = unformed or seedy, 5 = watery) was significantly lower in the Control versus PDX/GOS or GOS groups during study week 1 (3.1±0.0 vs 3.4±0.0 or 3.3±0.0; all *P* < 0.05) and study week 2 (3.0±0.1 vs 3.4±0.1 or 3.2±0.1; all *P* < 0.05). Stool consistency was also significantly lower in the GOS vs PDX/GOS group during study week 2 (*P* < 0.05). At 60 days, the distribution of stool consistency was significantly different between Control and PDX/GOS or GOS groups (Table 4; all *P* < 0.05). In stool consistency categories, the primary differences were more infants with a formed (13%) or soft (75%) and fewer infants with an unformed or seedy stool consistency (9%) in the Control group compared to PDX/GOS (formed, 6%; soft, 56%; unformed or seedy, 30%) and GOS groups (formed, 7%; soft, 67%; unformed or seedy, 22%). No significant group differences were detected at 90 days. At 120 days, again a significant difference in the distribution of stool consistency was detected between the Control and PDX/GOS groups (*P* < 0.05). The primary differences were a higher percentage of infants in the Control versus the PDX/GOS group with a formed (12% vs 5%) or soft (76% vs 68%) stool consistency and lower percentage with unformed or seedy (10% vs 18%) stool consistency.

**Table 4:** Stool characteristics at 14 (enrollment), 60, 90, and 120 days of age

**Age**	**Stool frequency**		**Overall*****P***	**Stool consistency, n (%)**					
	**Group (***n*****)	Mean (±SE)*		**hard**	**formed**	**soft**	**unformed or seedy**	**watery**	**Overall*****P***
14 days	Control (142)	2.6±0.2	0.074	4 (3)	6 (4)	88 (63)	40 (29)	2 (1)	0.526
	PDX/GOS (139)	3.0±0.2		5 (4)	10 (8)	66 (51)	44 (34)	5 (4)	
	GOS (138)	3.1±0.2		2 (2)	9 (7)	73 (55)	5 (4)	6 (5)	
60 days	Control (102)	2.0±0.1	0.002†‡	2 (2)	13 (13)	76 (75)	9 (9)	1 (1)	<0.001†‡
	PDX/GOS (101)	2.6±0.2		1 (1)	6 (6)	57 (56)	30 (30)	7 (7)	
	GOS (96)	2.6±0.2		0 (0)	7 (7)	64 (67)	21 (22)	3 (3)	
90 days	Control (99)	2.0±0.1	0.083	0 (0)	14 (14)	70 (71)	11 (11)	3 (3)	0.089
	PDX/GOS (97)	2.3±0.1		1 (1)	6 (6)	61 (66)	21 (23)	4 (4)	
	GOS (87)	2.4±0.1		0 (0)	6 (7)	64 (73)	15 (17)	3 (3)	
120 days	Control (97)	2.0±0.1	0.265	2 (2)	11 (12)	71 (76)	9 (10)	1 (1)	0.004†
	PDX/GOS (95)	2.3±0.1		0 (0)	5 (5)	63 (68)	17 (18)	7 (8)	
	GOS (87)	2.2±0.1		2 (2)	7 (8)	59 (70)	13 (15)	3 (4)	

* Mean ± standard error (SE) stool frequency. Means for 14, 60, 90, and 120 days are based on 24-hour recall at study visits.

†Control vs. PDX/GOS significantly different (*P <* 0.05).

‡Control vs. GOS significantly different (*P* < 0.05).

No group difference was detected in the number of participants for whom at least one medically-confirmed adverse event was reported (Control: 109, 78%; PDX/GOS: 97, 70%; GOS: 106; 77%). The incidence of adverse events categorized within Body as a Whole, Cardiovascular, Metabolic and Nutrition, Musculoskeletal, or Urogenital systems were generally low with no statistically significant group differences for specific events. Within the Eyes, Ears, Nose, and Throat system, the overall incidence of adverse events (Control: 43, 31%; PDX/GOS: 27, 19%; GOS: 43; 31%) was significantly lower in the PDX/GOS group versus either the Control or GOS groups (*P* < 0.05), however there were no significant differences between specific types of adverse events within this category. Within the Gastrointestinal (GI) System, the most commonly reported specific adverse events were gastroesophageal (GE) reflux, gas, emesis, and diarrhea. There were no group differences in the incidence of GE reflux, emesis, or diarrhea, however the incidence of gas (Control: 16, 11%; PDX/GOS: 4, 3%; GOS: 10, 7%) was significantly lower in the PDX/GOS versus the Control group (*P* < 0.05). Also within the GI System category, excessive spitting (Control: 0, 0%; PDX/GOS: 2, 1%; GOS: 7, 5%) was significantly lower in the Control versus the GOS group (*P* < 0.05). Within the Respiratory category, no significant group differences were detected for specific adverse events. Any medically-confirmed adverse event was considered serious if it met one or more of the following criteria: resulted in death, was life-threatening, required inpatient hospitalization or prolongation of existing hospitalization, resulted in persistent or significant disability/incapacity, or was a congenital anomaly/birth defect. A total of 21 participants experienced serious adverse events (Control: 7, 5%; PDX/GOS: 4, 3%; GOS: 10, 7%). All serious adverse events were individually evaluated by the study site physicians and each was determined to be unrelated to study formulas.

## Discussion

This study demonstrated that routine cow’s milk-based formulas supplemented with either a prebiotic blend of PDX and GOS or GOS alone were safe and well-tolerated when fed to healthy term infants from 14 to 120 days of age. Investigational formulas were also associated with normal growth throughout the study. The few differences detected between the control and investigational formula groups in body weight growth rates for males and females occurred only in the day 14 to 30 age range and were not accompanied by any statistically significant differences in length or head circumference growth rates. At 14 days all group means were between the 25^th^ and 50^th^ reference percentiles of the WHO weight growth chart. By 30 days all group means were near or above the 50th percentile and remained above the 50th percentile for the remainder of the study. In addition, there were no group differences detected for length or head circumference growth rates, weight growth rate within any other age range, or achieved values at any measured study time point. We have previously demonstrated that supplementation of PDX and GOS to routine, cow’s milk-based formula is well tolerated, safe, and promotes normal growth [9,12,20].

Each investigational study formula also included adjustments in total fat, total carbohydrate, level of ARA, and calcium source. The investigational formulas derived approximately 50% of total calories from fat (compared to 48% in the control, or 5.6 vs 5.3 g fat/100 kcal), in conjunction with a compensatory reduction in carbohydrates to approach the level and caloric contribution of fat typically found in human milk, which provides 50-60% of total calories and is not usually related to maternal dietary differences [21]. As expected, this slight adjustment in total fat did not result in differences in overall growth. In addition, DHA and ARA are the primary LCPUFAs found in human milk and both are always present in human milk, albeit at various concentrations and ratios [21-23]. Supplementation of both DHA and ARA (at ~0.3% and ~0.6% of total fat, respectively) to infant formulas based on previously published values for worldwide human milk [24,25] has been associated with visual and cognitive development in term infants [26-31] and enhanced growth and neural development in preterm infants [32,33]. A recent comprehensive, critical review of literature published on breast milk levels of DHA and ARA provided updated worldwide means for DHA at 0.32% (SD 0.21%; median 0.26%; mode 0.20%) and ARA at 0.47% (SD 0.13%; median 0.45%; mode 0.50%) of total fatty acids [23]. Consequently, we evaluated effects of the adjusted level of ARA supplementation to infant formula upon growth and safety. Finally, whereas all study formulas provided a final available calcium concentration of 78 mg/100 kcal, the success and safety of calcium gluconate used as one of several calcium sources in powdered preterm human milk fortifier [34] allowed us to evaluate this ingredient as the sole source of supplemental calcium within the matrix of a routine infant formula for growth and safety in term infants.

Overall, acceptance and tolerance of study formulas were good. No differences in study discontinuation due to study formula were detected. No differences in overall study discontinuation were detected and study discontinuation rates were as expected when compared to those reported in other large pediatric nutrition trials [35,36]. No significant differences were detected in fussiness or gassiness among study groups. Mean stool frequency was significantly lower in the Control versus either the PDX/GOS or GOS groups during study weeks 1 and 2 and at 60 days but no group differences were observed at 90 or 120 days. In this study, some significant differences in mean stool consistency were noted among groups during study weeks 1 and 2 and in stool consistency at days 60 and 120. Mean stool consistency was in the soft range for all study groups during study weeks 1 and 2, and the majority of infants in all groups at all measured time points were reported to have a soft stool consistency. However, more infants with formed stool consistency and fewer with unformed or seedy stool consistency were typically reported in the Control compared to the PDX/GOS or GOS groups. The stool softening effect demonstrated with the blend of PDX and GOS or GOS alone may potentially help manage hard stools that could affect formula-fed infants [37]. In general, softer, looser stools are characteristic of both breastfed infants and infants who receive formula supplemented with prebiotics when compared to those who receive unsupplemented formulas [11,38,39]. In healthy, term infants we previously reported that use of routine formulas supplemented with PDX and GOS produced a bifidogenic effect closer to breast milk compared to formula without PDX and GOS [12]. The current results are also consistent with our previous reports in which use of routine formulas supplemented with PDX and GOS produced softer stools in healthy, term infants compared to formula without PDX and GOS [9,12,20].

## Conclusion

Although breast milk is the ideal source of nutrition for infants, advancements in infant formula research are necessary to provide the best possible alternative for infants that cannot receive human milk. In this study, routine infant formula with adjustments in fat, carbohydrate, and calcium composition and supplemented with 4 g/L of either the prebiotic blend of PDX and GOS or GOS alone was well-tolerated and supported normal growth. Compared to infants who received the unsupplemented control formula, infants who received prebiotic supplementation experienced a softer stooling pattern similar to that reported in breastfed infants.

## Abbreviations

ARA, Arachidonic acid; DHA, Docosahexaenoic acid; GOS, Galactooligosaccharides; HMOs, Human milk oligosaccharides; PDX, Polydextrose.

## Competing interests

CA and WHJ have received research support from Mead Johnson Nutrition. CLB, CLH, SIS and JLW work in the Department of Medical Affairs at Mead Johnson Nutrition.

## Authors’ contributions

CA and WHJ assessed study participants and collected study data. CLB conceived of and designed the study. CLH participated in study design and performed statistical analyses. SIS participated in study design and study coordination. JLW participated in study design and drafted the manuscript. All authors interpreted data, contributed to the intellectual content, reviewed and revised the manuscript, and approved the final version.
